# Isothiocyanates induce UGT1A1 in humanized *UGT1* mice in a CAR dependent fashion that is highly dependent upon oxidative stress

**DOI:** 10.1038/srep46489

**Published:** 2017-04-19

**Authors:** Emiko Yoda, Miles Paszek, Camille Konopnicki, Ryoichi Fujiwara, Shujuan Chen, Robert H. Tukey

**Affiliations:** 1 Laboratory of Environmental Toxicology, Department of Pharmacology, University of California, San Diego, La Jolla, CA 92093, USA; 2 Division of Health Chemistry, Department of Healthcare and Regulatory Sciences, School of Pharmacy, Showa University, 1-5-8 Hatanodai, Shinagawa-ku, Tokyo 142-8555, Japan; 3 Department of Pharmaceutics, School of Pharmacy, Kitasato University, 6-9-1 Shirokane, Minato-ku, Tokyo 108-8641, Japan

## Abstract

Isothiocyanates, such as phenethyl isothiocyanate (PEITC), are formed following the consumption of cruciferous vegetables and generate reactive oxygen species (ROS) that lead to the induction of cytoprotective genes such as the UDP-glucuronosyltransferases (UGTs). The induction of ROS activates the Nrf2-Keap 1 pathway leading to the induction of genes through antioxidant response elements (AREs). UGT1A1, the sole enzyme responsible for the metabolism of bilirubin, can be induced following activation of Nrf2. When neonatal humanized *UGT1 (hUGT1*) mice, which exhibit severe levels of total serum bilirubin (TSB) because of a developmental delay in expression of the *UGT1A1* gene, were treated with PEITC, TSB levels were reduced. Liver and intestinal UGT1A1 were induced, along with murine CYP2B10, a consensus CAR target gene. In both neonatal and adult *hUGT1/Car*^−/−^ mice, PEITC was unable to induce CYP2B10. A similar result was observed following analysis of UGT1A1 expression in liver. However, TSB levels were still reduced in *hUGT1/Car*^−/−^ neonatal mice because of ROS induction of intestinal UGT1A1. When oxidative stress was blocked by exposing mice to N-acetylcysteine, induction of liver UGT1A1 and CYP2B10 by PEITC was prevented. Thus, new findings in this report link an important role in CAR activation that is dependent upon oxidative stress.

The consumption of cruciferous vegetables such as broccoli, watercress, and cauliflower have been correlated through epidemiological studies with a lower incidence of cancer development^1^,^2^,^3^. These vegetables are abundant in glucosinolates, which are hydrolyzed by plant myrosinase and by microflora of the gastrointestinal tract to form isothiocyanates (ITCs), commonly regarded as conveying active anticarcinogenic activity^4^,^5^. The anticarcinogenic activity of ITCs has been linked to the activation of the nuclear factor erythroid 2 related factor 2 (Nrf2)-Keap1 signaling pathway^6^, which is followed by binding of the transcriptional factor Nrf2 to antioxidant response elements (AREs)^7^,^8^. Prominent in this induction process are the cluster of genes that encode proteins involved in limiting both endogenous and exogenous chemicals from initiating deleterious biological effects by directing their metabolism, an event that biologically inactivates the agents and in many cases, facilitates their excretion from the body. This cumulative defense system, carried out by proteins that have been classified to participate in Phase II drug metabolism, prevents chemical induced toxic/genetic mutations and limits induction of epigenetic events that might lead to cellular proliferation and tumor development^9^.

The UDP-glucuronosyltransferase (UGT) family of proteins is part of the Phase II cluster and participates in the detoxification and genoprotective process by converting target substrates to water soluble products by glucuronidation^10^,^11^. Once the glucuronides are formed, the soluble metabolites are transported out the cell, in many instances through high affinity transporters^12^,^13^,^14^, and become sequestered in the water compartments of the tissue that eventually lead to elimination. Thus, the UGTs play a key role in detoxification of many endogenous agents, such as steroids and bile acid, hundreds of therapeutically consumed drugs, and environmental toxicants that are known mutagens and carcinogens^11^. The substrate specificity attributed to UGTs occurs in part because of a large and diversified gene family of proteins. In human, 19 UGTs have been identified, with each being classified into the UGT1 (UGT1A1, -1A3, -1A4, -1A5, -1A6, -1A7, -1A8, -1A9 and -1A10) or UGT2 family (-A1, -A2, -A3, -B4, -B7, -B10, -B11, -B15, -B17 and -B28)^15^. Each of the *UGT* genes is dynamically regulated, as evident from unique expression patterns observed in human tissues^11^. When human tissues were examined to document gene expression patterns, each tissue displayed a unique complement of the UGTs, providing evidence that tight tissue specific regulation leads to diversified glucuronidation activity^16^,^17^,^18^,^19^.

Cellular and molecular events that underlie the regulation of the UGTs, especially those encoded by the human *UGT1* gene family, have been studied in humanized transgenic mice that express the human *UGT1* locus in a *Ugt1*-null background (*humanized UGT1* mice)^20^. Developmental and tissue specific regulation of *UGT1A* genes has been shown to reflect similar patterns of expression as characterized in human tissues^21^. For example, the human *UGT1A1* gene, which is developmentally delayed in newborns^22^, is also developmentally repressed in *hUGT1* neonatal mice^23^. Since UGT1A1 is the sole enzyme responsible for the metabolism of serum bilirubin^24^, this delay in UGT1A1 expression leads to severe neonatal hyperbilirubinemia in *hUGT1* mice. The *UGT1A1* gene can be transcriptionally induced by a host of xenobiotic nuclear receptors, including the pregnane X receptor (PXR)^25^, the constitutive androstane receptor (CAR)^26^, the peroxisome proliferator-activated receptor alpha (PPARα)^27^, the liver X receptor (LXR), and the environmental sensors, Ah receptor^28^,^29^ and Nrf2^30^. The oral or intraperitoneal administration of ligands capable of activating these receptors in neonatal *hUGT1* mice leads to the induction of liver or intestinal UGT1A1 followed by dramatic reductions in total serum bilirubin (TSB) levels. Induction of UGT1A1 following activation of the Nrf2-Keap1 signaling pathway by oxidants, such as tert-butylhydroquinone, implicates a potential role for the AREs responding to oxidative stress in control of the *UGT1A1* gene^30^.

It is generally believed that ITCs, such as phenethyl isothiocyanate (PEITC), induce UGT1A1 through activation of the Nrf2-Keap1 pathway, since the *UGT1A1* gene is a known target of Nrf2^30^. We have extended these findings by treating neonatal and adult *hUGT1* mice with PEITC and have evaluated the impact of ITCs *in vivo* for its capability to reduce serum bilirubin in neonatal mice while inducing a genome wide array of potential target genes. We will demonstrate that along with Nrf2 response genes, such as *Nqo1* being significantly induced in adult hepatic tissue, CAR dependent target genes, such as *Cyp2b10*, are also upregulated. In addition, evidence will be presented linking the generation of reactive oxygen species (ROS) by PEITC in neonatal intestinal tissue to activation of intestinal epithelial cell maturation, a process that was recently shown to be linked to derepression of the nuclear corepressor protein NCoR1 and induction of intestinal UGT1A1^31^. These observations led us to examine in greater detail the potential cross-talk between the PEITC generated antioxidant response and CAR activation, and the implications of ROS on activation and induction of UGT1A1.

## Materials and Methods

### Chemical reagents

Phenethyl isothiocyanate (PEITC) and N-acetyl-L-cysteine (NAC) were obtained from Sigma-Aldrich (St. Louis, MO). Rabbit anti-UGT1A1 monoclonal antibody was purchased from abcam (Cambridge, UK). Mouse anti-GAPDH monoclonal antibody and rabbit anti-CYP2B9/10 antibody were obtained from Santa Cruz Biotechnology (Dallas, TX). Anti-p-p38, anti-p38, anti-mouse IgG horseradish peroxidase (HRP) conjugated antibody and anti-rabbit IgG HRP conjugated antibodies were obtained from Cell Signaling Technology, Inc. (Danvers, MA).

### Animals

Transgenic mice expressing the human *UGT1* locus (UGT1A1*1) in a *Ugt1*^−/−^ background (*hUGT1*)^32^ were developed previously^20^. *hUGT1/Car*^−/−^ mice were constructed previously^23^. All animals were housed at the University of California San Diego (UCSD) Animal Care Facility and received food and water ad libitum. All animal use protocols, mouse handling, and experimental procedures were approved by the UCSD Animal Care and Use Committee (IACUC), and these protocols were conducted in accordance with federal regulations.

For neonatal studies, 10-day-old *hUGT1, hUGT1/Car*^+/−^ or *hUGT1/Car*^−/−^ mice were treated by oral gavage with corn oil (vehicle) or 200 mg/kg PEITC dissolved in vehicle. Blood and tissues were collected as indicated in the figures. For adult studies, vehicle or PEITC (200 mg/kg) was orally administrated to 6-week-old male mice every day for 4 days and tissues were collected on the 5th day after PEITC administration.

### Administration of PEITC, arsenic and antioxidant NAC

Adult *hUGT1* male mice (3 weeks old) were divided into 4 groups (n = 3–4 per group): control + vehicle group was given drinking water for 3 weeks and then administrated vehicle (corn oil) orally for 4 days; control + PEITC group was given drinking water for 3 weeks and then PEITC (200 mg/kg) was administrated orally for 4 days; N-acetyl cysteine (NAC) + vehicle group was given 40 mM NAC in drinking water for 3 weeks and then vehicle (corn oil) was administrated orally for 4 days; NAC + PEITC group was given 40 mM NAC in drinking water for 3 weeks and PEITC (200 mg/kg) was administrated once a day for 4 days by oral gavage. Tissues were isolated five days after vehicle or PEITC administration.

Neonatal *hUGT1* mice (10 days old) were given an oral dose of NAC (75 mg/kg) 1 hour prior to the oral administration of arsenic (As^3+^) at a dose of 10 mg/kg. Forty-eight hours later TSB levels were taken and tissue was collected. For PEITC treatment, neonatal mice were given an oral dose of 200 mg/kg and TSB levels measured after 48 hours and tissues collected.

### Bilirubin measurements

A few drops of blood were obtained from the submandibular vein and centrifuged at 2,000 × g for 5 min. Serum samples were immediately measured for total serum bilirubin (TSB) levels using a Unistat Bilirubinometer (Reichert, Inc.).

### Reverse Transcription Quantitative-PCR

Tissue samples were homogenized in 0.5 mL TRIzol (Invitrogen, Waltham, MA) and total RNA from whole tissues was isolated using TRIzol reagents per the manufacturer’s instructions. Using iScript Reverse Transcriptase (Bio-Rad Laboratories, Hercules, CA), 1 μg of total RNA was used for the generation of cDNA in a total volume of 20 μL as outlined by the manufacturer. Following cDNA synthesis, quantitative PCR was carried out on a CFX96 qPCR system (BioRad) by using SsoAdvanced SYBR Green Supermix (BioRad). Primers were designed through the mouse primer depot (https://mouseprimerdepot.ncimci,.nih.gov/). The sequences of the primers used are listed in Table 1.

### Western blot analysis

Tissues (0.1 mg) were homogenized in 0.5 mL 1 × RIPA lysis buffer (EMD Millipore, Billerica, MA) supplemented with protease inhibitor cocktail (Sigma-Aldrich). Western blots were performed by using NuPAGE 4–12% BisTris-polyacrylamide gels (Invitrogen) with the protocols described by the manufacturer. Protein (20 μg) was electrophoresed at 200 V for 50 min and transferred at 30 V for 2 hours to PVDF membranes (EMD Millipore). Membranes were blocked with either 5% skim milk or 5% BSA (fraction V) at room temperature for 1 hour and incubated with primary antibodies (rabbit anti-human UGT1A1 (ab 194697), mouse anti-GAPDH (sc-47724), rabbit anti-CYP2B9/10 antibody, rabbit anti-p-p38 (cs-9211) or rabbit anti-p38 (cs-9212)) at 4 °C overnight. Membranes were washed and exposed to HRP-conjugated secondary antibodies (anti-mouse IgG or anti-rabbit IgG) for 1 hour at room temperature. Protein was detected by the ECL Plus Western blotting detection system (PerkinElmer) and was visualized by the BioRad gel documentation system. All Western blots are cropped from the full length blots that have been included in the Supplemental Material.

### Statistical analysis

Statistical significances were determined by analysis of variance by using Student’s t test. *P* values < 0.05 were considered statistically significant, and statistically significant differences are indicated with *P < 0.05; **P < 0.01; ***P < 0.001.

## Results

### Impact of PEITC in neonatal *hUGT1* mice

**Figure 1:**
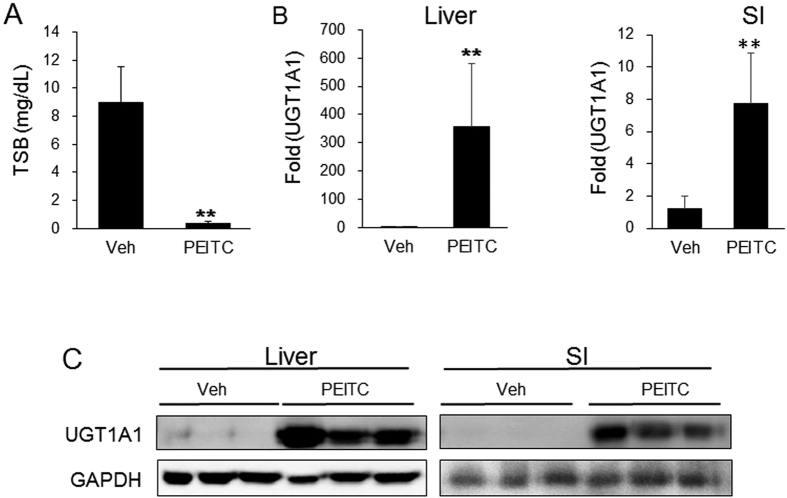
Induction of UGT1A1 by PEITC in neonatal hUGT1 mice. Ten day old neonatal *hUGT1* mice were treated with 200 mg/kg PEITC or vehicle by p.o. administration. (**A**) Forty eight hours after the treatment, total serum bilirubin (TSB) levels were taken. (**B**) Liver and small intestine (SI) were pulverized under liquid nitrogen and used for the preparation of total RNA or whole tissue extracts. From RNA preparations, *UGT1A1* gene expression was determined by real time PCR and expressed as fold induction. (**C**) Using whole tissue extracts, Western blots were performed from liver and SI tissue to examine UGT1A1 expression and imaged on a BioRad ChemiDoc Touch Imaging System. The bands have been cropped from the full-length blots but not enhanced in anyway (**S4**). Values are the means of ±SD (n > 3). Statistically significant differences between vehicle (Veh) and PEITC are indicated by asterisks (Student *t* test: **P < 0.01).

**Figure 2:**
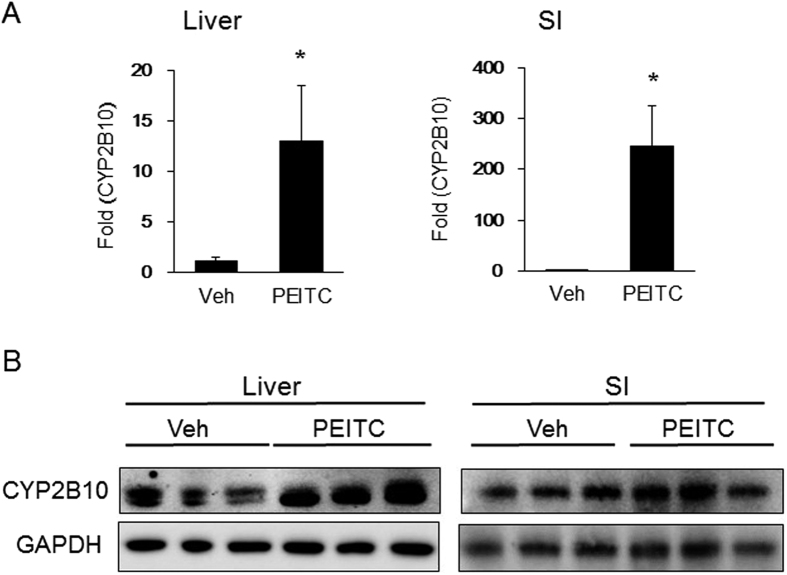
PEITC induction of CYP2B10 in neonatal hUGT1 mice. Following the treatment of neonatal *hUGT1* mice with 200 mg/kg PEITC for 48 hours (see Fig. 1), *Cyp2b10* gene and CYP2B10 protein expression were analyzed. (**A**) Using RNA isolated from liver and small intestines (SI), mRNA induction was quantitated by real time PCR. (**B**) Total tissue extracts prepared from the same tissues were used for Western blot analysis to examine CYP2B10 expression. The bands have been cropped from the full-lenth blots but not enhanced in anyway (**S5**). Values are the means of ±SD (n > 3). Statistically significant differences between vehicle (Veh) and PEITC are indicated by asterisks (Student *t* test: *P < 0.05).

We have shown previously that xenobiotic induction of either gastrointestinal (GI) tract or liver UGT1A1 will promote the reduction of total serum bilirubin (TSB) in neonatal *hUGT1* mice^20^,^33^. The impact of oral PEITC treatment to neonatal mice would be expected to induce ARE target genes both in the GI tract as absorption is occurring as well as in the liver prior to movement of PEITC into the systemic circulation. After a single oral dose of 200 mg/kg to 10-day-old *hUGT1* mice, TSB levels were reduced to normal levels after 48 hours (Fig. 1A), indicating that UGT1A1 was induced. Analysis of gene expression and protein in small intestine (SI) and liver shows induction of UGT1A1 (Fig. 1B,C). Target genes of the antioxidant response, *Nqo1, Gsta1* and *Gsta2* were not induced dramatically in either tissue (**S1**), indicating that an alternative mechanism other than an antioxidant response was underlying the induction of UGT1A1. Analysis of additional potential gene expression differences that might exist between liver and SI following PEITC administration confirmed induction of the *Cyp2b10* gene and protein expression in both tissues (Fig. 2), an observation that implicates a role for CAR in the induction process.

**Figure 3:**
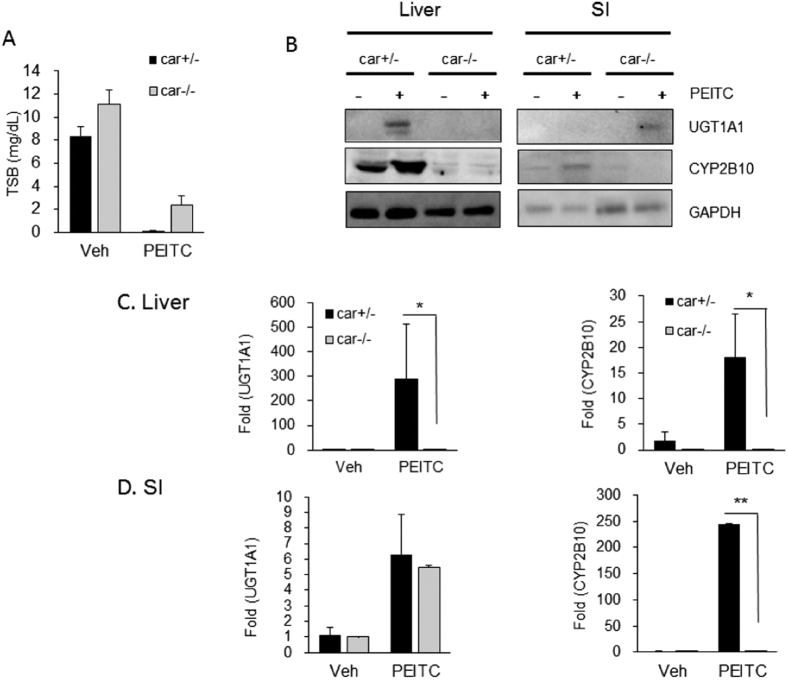
PEITC induction in hUGT1/Car-null mice. Litters were bred to produce *hUGT1/Car*^−/−^ and *hUGT1/Car*^*+/−*^ mice and 10-day old neonatal mice treated with 200 mg/kg on day 10, followed by TSB analysis and tissue preparation on day 12. (**A**) After treatment, TSB levels were quantitated in *hUGT/Car*^*+/−*^ (car^+/−^) and *hUGT1/Car*^−/−^ (car^−/−^) mice. (**B**) Using total cell extracts from liver and small intestines (SI), Western blots were performed using antibodies toward human UGT1A1 and mouse CYP2B10. The bands have been cropped from the full-length blots but not enhanced in anyway (**S6**). (**C**) Total RNA was prepared from liver and small intestines and mRNA expression determined by real time PCR. Values are the means of ±SD (n > 3). Statistically significant differences between vehicle (Veh) and PEITC are indicated by asterisks (Student *t* test: *P < 0.05; **P < 0.01).

To examine the possibility that CAR is playing a role following exposure to PEITC, 10-day-old *hUGT1/Car*^−/−^ mice were treated orally with PEITC and analysis of both TSB levels, UGT1A1 and CYP2B10 expression were monitored in both liver and SI (Fig. 3). TSB levels were completely reduced in *hUGT1/Car*^+*/*−^ mice and significantly reduced in *hUGT1/Car*^−/−^ litter mates but were not as low as those observed in *hUGT1/Car*^*+/−*^ mice (Fig. 3A). Deletion of CAR led to the complete lack of PEITC initiated induction of CYP2B10 and UGT1A1 expression in liver (Fig. 3B), confirming that PEITC exposure activates liver CAR. When we examined SI, CYP2B10 expression was absent in *hUGT1/Car*^−/−^ mice, but induction of UGT1A1 was observed (Fig. 3B). When gene expression patterns were examined in liver, induction of *Cyp2b10* and *UGT1A1* gene expression was absent in *hUGT1/Car*^−/−^ mice, confirming the role of CAR in activation of these genes in hepatic tissue (Fig. 3C). Intestinal tissue showed a different induction pattern. The *Cyp2b10* gene was regulated in concordance with PEITC activation of CAR, showing no activation of the gene in *hUGT1/Car*^−/−^ mice. In contrast, PEITC initiated induction of *UGT1A1* gene expression equally in the SI of both *hUGT1/Car*^*+/−*^ and *hUGT1/Car*^−/−^ mice (Fig. 3D), a finding that would suggest the induction of UGT1A1 in intestinal tissue is being controlled by an alternate mechanism that is not linked to CAR activation.

**Figure 4:**
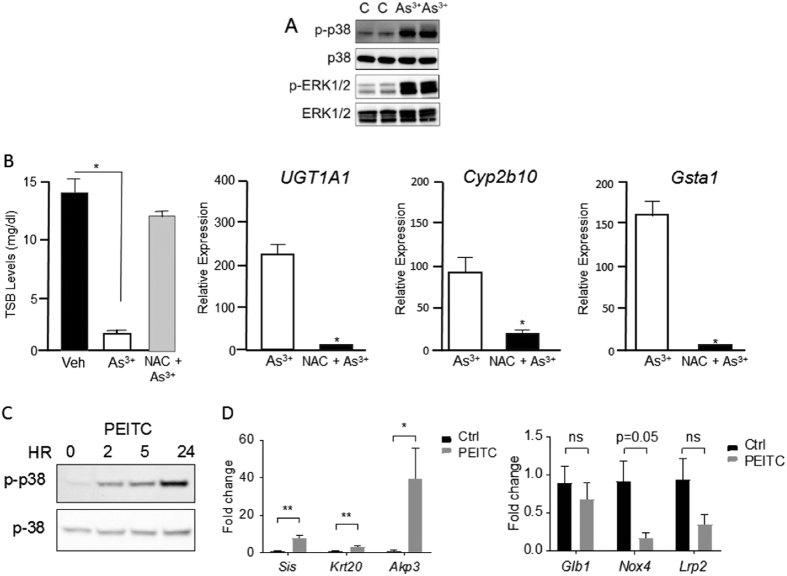
Induction of oxidative stress induces UGT1A1 in intestinal tissue. Neonatal hUGT1 mice were given a dose of either arsenic (As^3+^) or PEITC and markers of oxidative stress determined. (**A**) Ten day old mice were given a single oral dose of As^3+^ (10 mg/kg) and tissue extracts from the small intestine prepared after 4 hours. Western blots were prepared and phosphorylated p38 MAPK and ERK determined. The bands have been cropped from the full-length blots but not enhanced in anyway. (**B**) Ten day old mice were pre-treated with 75 mg/kg N-acetylcysteine (NAC) for one hour prior to As^3+^ exposure. Forty-eight hours after oral As^3+^ exposure, RNA was isolated from small intestine and RT-qPCR was conducted for *UGT1A1, Cyp2b10* and *Gsta1* expression. Data is shown as fold induction of the value observed in mice treated with As^3+^ and water. (**C**) Ten-day-old mice were given an oral dose of PEITC (200 mg/kg) and intestinal tissue isolated at 0, 2, 5, and 24 hours after treatment. Western blots were performed to examine activation of phosphorylated p38 MAPK (**S7**). (**D**) Twenty-four hours after PEITC treatment, RNA was prepared from small intestines and RT-qPCR performed to examine intestinal maturation marker gene expression. Genes *Sis, Akp3* and *Krt20* are upregulated during maturation while genes *Glb1, Nox4* and *Lrp2* are downregulated (Student t test: ^*^P < 0.05; ^**^P < 0.01).

To examine this possibility in greater detail, we undertook a series of experiments to determine if an oxidative stress response could be induced in the intestines. We have shown previously that oral arsenic (As^3+^) exposure to neonatal *hUGT1* mice induce *Cyp2b10* and *UGT1A1* gene expression^33^. If we treat neonatal *hUGT1* mice with As^3+^ and measure phosphorylated p38 mitogen-activated protein kinase (MAPK) and extracellular signal-regulated kinase (ERK) activation, both markers of oxidative stress^34^, induction is observed as early as 2 hours after treatment (Fig. 4A). N-acetylcysteine (NAC), which serves as a precursor to elevate intracellular GSH^35^, blocks the effects of oxidative stress. We treated 10-day old *hUGT1* mice with oral NAC and then challenged the mice with oral As^3+^, measuring gene expression after 24 hours. Arsenic treatment lead to a dramatic reduction in TSB levels, that was blocked when the mice were pretreated with NAC. Arsenic induced *UGT1A1, Cyp2b10* and *Gsta1* gene expression, all of which were inhibited by NAC treatment (Fig. 4B). When we measured phosphorylated p38 MAPK after PEITC treatment as an index of oxidative stress, there was a rapid and sustained activation of p38 MAPK even through 24 hours (Fig. 4C), demonstrating that PEITC elicited a strong oxidative stress response in intestinal tissue. This was also verified by an increase in GSSG in intestinal tissue (**S2**). However, this response was not sufficient to activate Nrf2 target genes (**S1**).

It has recently been demonstrated that nuclear receptor corepressor 1 (NCoR1) in intestinal epithelial tissue controls neonatal intestinal maturation by repressing gene expression^31^. When NCoR1 is deleted by targeted gene interruption in intestinal tissue, epithelial cell maturation is accelerated in neonatal *hUGT1* mice resulting in derepression of *UGT1A1* gene expression with the near complete reduction in TSB levels. Intestinal epithelial cell (IEC) maturation can be analyzed by measurement of IEC specific maturation markers, such as the upregulated *Sis, Akp3*, and *Krt20* genes, and the downregulated *Glb1, Nox4* and *Lrp2* genes. Intestinal maturation can be induced by activated cellular kinases, which are believed to target phosphorylation of NCoR1 leading to derepression of gene expression^36^. Since PEITC is a potent inducer of intestinal p38 MAPK, we examined these maturation marker genes (Fig. 4D). Regulation of these genes followed closely the patterns of expression that were documented during neonatal intestinal maturation by expressed IKKβ and following targeted deletion of NCoR1^31^. The possibility exists that the sharp increase in oxidative stress by PEITC promotes intestinal maturation, leading to derepression of the *UGT1A1* gene and the reductions we have observed in TSB levels.

### Impact of PEITC in adult *hUGT1* mice

**Figure 5:**
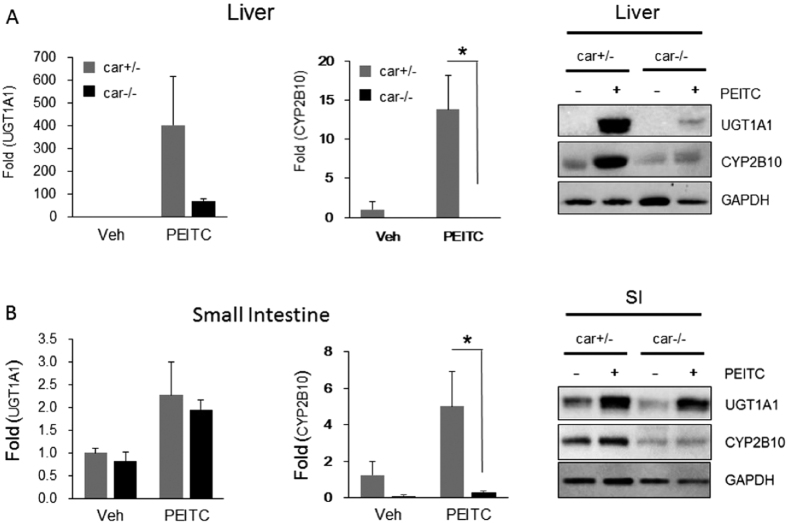
Induction by PEITC in adult hUGT1/Car^−/−^ mice. Adult (8 week) *hUGT1/Car*^−/−^ and *hUGT1/Car*^*+/−*^ mice were treated by p.o administration with 200 mg/kg/day for four days. After treatment, RNA and total cell extracts were prepared. (**A**) From liver tissue, *UGT1A1* and *Cyp2b10* gene expression was analyzed by real time PCR and expressed as Fold induction. From the same tissues, total cell extracts were combined and used for Western blot analysis using anti-human UGT1A1 and anti-mouse CYP2B10 antibodies. The bands have been cropped from the full-length blots but not enhanced in anyway (**S7**). (**B**) From small intestine, a similar series of experiments were performed. Values are the means of ±SD (n > 3). Statistically significant differences between vehicle (Veh) and PEITC are indicated by asterisks (Student *t* test: *P < 0.05).

To examine the association of CAR activation and the anti-oxidative response in adult mice, we treated *hUGT1/Car*^*+/−*^ and *hUGT1/Car*^−/−^ mice orally with PEITC and evaluated gene and protein expression in liver and SI (Fig. 5). As observed in neonatal *hUGT1* mice, PEITC led to induction of *UGT1A1* and *Cyp2b10* gene expression in liver as well as SI in *hUGT1/Car*^*+/−*^ mice. Protein expression paralleled the findings of gene expression, with Western blots confirming induction of UGT1A1 and CYP2B10 in liver and SI. The induction of CYP2B10 in liver is completely dependent upon CAR, since no induction was noted in *hUGT1/Car*^−/−^ mice (Fig. 5A). There was also a dramatic reduction in the induction of UGT1A1 in *hUGT1/Car*^−/−^ mice, implicating an important role for CAR in PEITC activation. However, we did detect both at the mRNA and protein level minor induction of UGT1A1 in *hUGT1/Car*^−/−^ mice, indicating that an alternative mechanism exists. Analysis of Nrf2 target genes confirmed induction was taking place by an oxidative stress response (**S3**). In the SI, PEITC treatment led to induction of the *UGT1A1* gene and protein expression in both *hUGT1/Car*^*+/−*^ and *hUGT1/Car*^−/−^ mice (Fig. 5B). We have confirmed that PEITC does activate CAR in the SI, as shown by CAR dependent activation of the *Cyp2b10* gene. While *UGT1A1* is also a target following CAR activation by PEITC, the ability to induce UGT1A1 in *hUGT1/Car*^−/−^ mice indicates that in this tissue the actions of PEITC are activating *UGT1A1* gene expression and protein induction through an alternative signaling pathway. Since ARE genes are induced in the SI following treatment with PEITC (**S3**), it is reasonable to assume that an antioxidant response by PEITC is initiating induction of UGT1A1 in the SI.

### The role of oxidative stress and control of CAR

**Figure 6:**
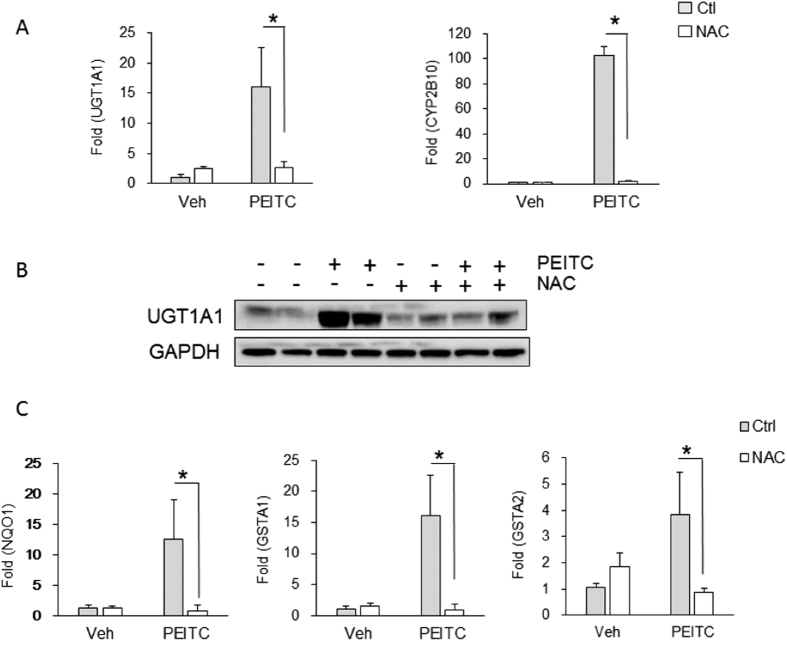
Impact of N-acetylcysteine on PEITC induction of UGT1A1. N-acetylcysteine (NAC) was added to the drinking water at a concentration of 40 mM for 3 weeks. After NAC exposure, adult *hUGT1* mice were treated by p.o. administration with 200 mg/kg PEITC once each day for four days. Liver was isolated after PEITC treatment. (**A**) From total RNA, human *UGT1A1* and mouse *Cyp2b10* gene expression was determined by real time PCR. (**B**) Using total cellular lysates, UGT1A1 expression was determined by Western blot analysis. The bands have been cropped from the full-length blots but not enhanced in anyway (**S8**). (**C**) *Nqo1, Gsta1* and *Gsta2* gene expression by real time PCR. Values are means ± SD (n = 3). Statistically significant differences between Vehicle (Veh) and PEITC are indicated by asterisks (Student *t* test: *P < 0.05)

To separate an anti-oxidative response initiated by PIETC from activation of CAR and induction of UGT1A1, we first administered adult *hUGT1* mice N-acetylcysteine (NAC) in their drinking water for 3-weeks. NAC prevents oxidative damage in tissues and cells by altering the redox-state and provides a reducing environment by increasing intracellular glutathione concentrations^35^. After two weeks of NAC exposure, *hUGT1* mice were administered PIETC. Examining liver, anti-oxidant-generated gene expression patterns for *Nqo1, Gsta1* and *Gsta2*, which were induced by PIETC, were blocked following NAC exposure (Fig. 6). Similarly, induction of gene expression for both liver *UGT1A1* and *Cyp2b10* by PEITC was inhibited because of NAC treatment (Fig. 6B). For UGT1A1 expression, NAC exposure completely blocked PEITC induction of UGT1A1. These results indicate that by replenishing intracellular antioxidant glutathione levels, NAC blocked PIETC-medicated antioxidant responses, and more surprisingly, abolished induction of both *Cyp2b10* and *UGT1A1* gene expression. These findings strongly suggesting that the Nrf2-ARE signaling pathway is actively involved in regulating these genes. Since we have demonstrated in liver that induction of the *Cyp2b10* and *UGT1A1* genes is driven by activation of CAR following PIETC exposure, this finding indicates that there is an important association between the production of ROS by PIETC and activation of CAR.

## Discussion

The antioxidant initiated induction patterns following exposure to PEITC have been shown to result from activation of the transcription factor Nrf2 that leads to its binding to ARE elements followed by transcriptional activation^37^,^38^,^39^. Activation of the Nrf2-Keap1 pathway in response to chemical and oxidative stress leads to the induction of a host of cytoprotective enzymes that provide an active defense mechanism against cellular damage. This unique signaling pathway was first discovered when ARE sequences were identified on two genes that encode enzymes involved in detoxification processes, NQO1^40^ and GSTA2^41^. The *Nqo1* and *Gsta2* genes are induced through the ARE elements following exposure to H_2_O_2_ and other agents that undergo redox cycling, or by compounds that are electrophilic. Other agents, such as the isothiocyanate PEITC, react with sulfhydryl groups to elevate the levels of ROS and reduce the antioxidant capacity, which in turn trigger the transcriptional response mediated by the AREs. Other genoprotective genes associated with cellular detoxification processes, such as UGTs, have long been implicated as important target genes that are induced following chemical and oxidative stress. A cluster of ARE elements in the phenobarbital response enhancer module (PBREM) in the *UGT1A1* gene has been identified and promotes Nrf2 dependent transcriptional activation^30^. Based upon these findings, it would be an obvious assumption that induction of the human *UGT1A1* gene following PEITC exposure would result from the production of an antioxidant response.

The previous development of *hUGT1* mice offered us the opportunity to examine activation of the *UGT1A1* gene since chemicals or agents administered to neonatal *hUGT1* mice that result in the reduction of TSB levels are directly inducing UGT1A1 expression^20^,^23^,^33^,^42^,^43^. When PEITC was administered orally to neonatal *hUGT1* mice, within 48 hours the TSB levels were reduced to those measured in wild type mice. Induction of *UGT1A1* gene and protein expression is significant in both SI and liver, indicating that a prominent antioxidant response was initiated following PEITC dosing. However, there was no significant induction of other key antioxidant target genes related to the Nrf2 signaling pathway, such as *Nqo1, Gsta1* or *Gsta2*, leaving us to speculate that induction of UGT1A1 was occurring independently from a Nrf2-medicated antioxidant response in the neonatal mice, although we would not rule out the possibility that Nrf2 is not fully activated at our experimental dose. Examination of additional gene expression patterns confirmed that PEITC dramatically induced *Cyp2b10* gene and protein expression in both SI and liver, an important target gene that is regulated following activation of CAR. When we extended these experiments to examine the impact of oral PEITC on TSB levels and UGT1A1 expression in neonatal *hUGT1/Car*^−/−^ mice, TSB levels were reduced. However, there was no induction of UGT1A1 or CYP2B10 in liver tissue, confirming that PEITC induction of the *UGT1A1* and *Cyp2b10* genes in neonatal *hUGT1* mice is CAR dependent. While intestinal *Cyp2b10* gene expression is absent in neonatal *hUGT1/Car*^−/−^ mice, we still observed induction of *UGT1A1* gene expression, leaving us to conclude that the actions of PIETC and induction of UGT1A1 in SI were occurring independently of CAR.

To examine this possibility, we first looked at the possibility of As^3+^, a known activator of oxidative stress and potent inducer of neonatal UGT1A1 and CYP2B10^33^, to activate stress linked proteins such as p38 MAPK. Oral As^3+^ rapidly activated phosphorylated p38 MAPK, demonstrating that oxidative stress was induced. When this process was blocked by pre-treatment with NAC, induction of *UGT1A1* gene expression and the reduction in TSB values were blocked. This experiment indicated that activation of cellular kinases by oxidative stress may contribute to induction of intestinal UGT1A1. When we administered PEITC to neonatal *hUGT1* mice, intestinal phosphorylated p-38 MAPK was dramatically activated at least through 24 hours. Recent experiments conducted in our laboratory have confirmed that derepression of NCoR1 in neonatal intestinal tissue results in activation of intestinal maturation and the induction of UGT1A1^31^. This process may be linked to the actions of PEITC since the mechanism that leads to NCoR1 derepression requires direct phosphorylation^44^, an event that may be linked to PEITC activated p38 MAPK. This possibility is supported by analysis of maturation gene expression profiles, an event that we have shown leads to induction of intestinal UGT1A1 and a reduction in TSB levels^31^.

In adult mice, PEITC administration led to prominent induction of UGT1A1 and CYP2B10 in liver and SI tissue, but there was a clear dominance of the induction pattern in liver. In contrast to induction patterns characterized in neonatal *hUGT1* mice, PEITC treatment to adult mice led to significant induction of an antioxidant response as confirmed by induction of target genes *Nqo1, Gst1a* and *Gsta2*. However, deletion of CAR in adult *hUGT1/Car*^−/−^ mice resulted in the elimination of both *UGT1A1* and *Cyp2b10* gene induction by PEITC, confirming previous findings that induction of these genes is dependent upon CAR. Collectively, these results indicate that PEITC has dual effects on activating both CAR and Nrf2, conveying its antioxidant activity by inducing cellular detoxification processes – including the phase I *Cyp2b10* and phase II, *UGT1A1, Nqo1, Gsta1* and *Gsta2* genes. These genes are regulated in an age- and tissue-specific fashion by PEITC with different modes of actions: Following PEITC treatment, CAR appears to be the dominant regulator in CYP2B10 induction, whereas activation of both CAR and Nrf2 is associated with UGT1A1 induction. In line with these results is the evidence that the PBREM but not the ARE region has been identified in the *Cyp2b10* promoter^45^, although it has recently been suggested that Nrf2 is involved in phorone induction of *Cyp2b10*^46^. In contrast, both response elements have been proven to be functional and located adjacent to each other within *UGT1A1* promoter sequences^30^. In addition, in neonates CAR has a preferential role in regulating UGT1A1, and the Nrf2-antioxidant pathway becomes more responsive to PEITC as mice reach maturity. Although liver and SI tissues generally exhibit similar induction patterns responding to PEITC exposure, our results suggest that induction of hepatic UGT1A1 is predominantly regulated by CAR, and its expression in SI may be attributed to both CAR and Nrf2.

**Figure 7:**
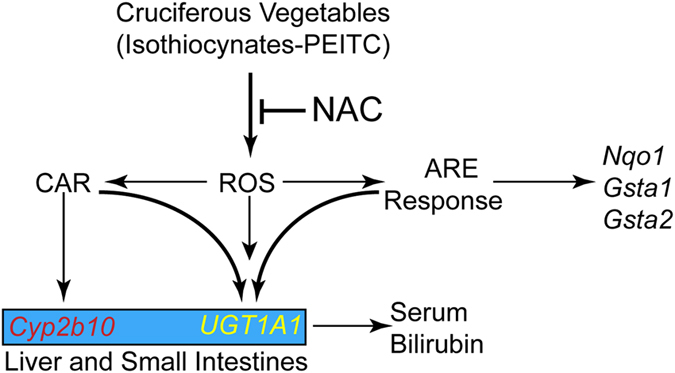
The actions of PIETC and regulation of UGT1A1 gene expression. Consumption of cruciferous vegetables leads to the generation of reactive oxygen species (ROS). The ROS generates signals that leads to the activation Nrf2, facilitating an ARE response on susceptible target genes. In addition, ROS are linked to the activation of CAR in both liver and small intestines, which leads to the induction of Cyp2b10 and UGT1A1. In neonatal mice, it is also predicted that ROS have the potential to induce intestinal tissue maturation, an event that has been shown to induce the *UGT1A1* gene. The induction of UGT1A1 in either the liver or small intestine will promote the metabolism and elimination of serum bilirubin. The addition of N-acetylcysteine (NAC) blocks the ROS response, nullifying both CAR and Nrf2 activation.

To isolate the antioxidant response from CAR dependency in PEITC induction of UGT1A1 and CYP2B10, we exposed adult *hUGT1* mice to NAC to block PEITC induced ROS production. Addition of the free radical scavenger NAC led to not only the complete inhibition of PEITC-induced expression of antioxidant genes *Nqo1, Gsta1* and *Gsta2* but also the blockage of CAR-initiated induction of the *UGT1A1* and *Cyp2b10* genes. This finding is significant because it indicates PEITC is the source of ROS enhanced expression of the *UGT1A1* and *Cyp2b10* genes through a ROS-dependent mechanism, further supporting the involvement of redox-sensitive transcription factor Nrf2 in controlling expression of UGT1A1 and CYP2B10. In combination with our findings that CAR is essential for PEITC initiated induction of UGT1A1, NAC blocks the PEITC induced antioxidant response, impacting induction of *UGT1A1* and *Cyp2b10* gene expression, suggesting an interaction between PEITC-produced oxidative stress and CAR activation, however the mechanism underlying the crosstalk between CAR and Nrf2 still needs to be determined. An outline of the proposed mechanism of PEITC initiated induction of the *Cyp2b10* and *UGT1A1* genes in liver and small intestine is outlined in Fig. 7.

In adult *hUGT1* mice, PEITC exposure produces an antioxidant response in both liver and SI, but the same response is not achieved in neonatal *hUGT1* mice. Bilirubin is a natural antioxidant when bound to albumin^47^ and slightly elevated levels in individuals that inherit Gilbert’s syndrome have been documented to have reduced rates of cardiovascular disease in addition to certain cancers^48^. Hence, the elevated concentrations of TSB in neonatal *hUGT1* mice may be sufficient to prevent PEITC induced ROS production, which is necessary to initiate an antioxidant response. When maternal mice were administered a daily dose of PEITC, nursing pups demonstrated a reduction in TSB levels (data not shown). The reduction in TSB levels correlated with induction of intestinal UGT1A1 expression. These findings indicate that neonatal hyperbilirubinemia can possibly be controlled through a maternal diet.

Our studies indicate that certain dietary ingredients present in cruciferous vegetables have a beneficial effect by providing a reductive capacity in tissues while also controlling important detoxification pathways such as glucuronidation, which are controlled at the transcriptional level through multiple signaling pathways. In addition to the important antioxidant response that leads to activation of the Nrf2-Keap1 pathway, we show here that ITCs regulate significant gene expression through a CAR dependent mechanism. However, the CAR dependent induction process, which we show exists for both the human *UGT1A1* gene and the murine *Cyp2b10* gene, appears to be linked mechanistically with a controlled antioxidant response. This relationship between CAR and Nrf2, which are both activated by PEITC, may serve as a double-edged-sword with regards to liver disease. Under these circumstances, agents that activate CAR stimulate the proliferative process and serve as tumor promotors^49^. Recently, it has been demonstrated that the accumulation of p62, a protein that can activate multiple signaling pathways, is induced in liver disease such as liver fibrosis and HCC. Induction of p62 leads to the activation of Nrf2, and sustained Nrf2 activation has been shown to be oncogenic^50^. Thus, in situations of liver disease, a diet rich in cruciferous vegetables would promote tumorigenesis through the activation of CAR while stimulating additional oncogenesis by activation of Nrf2. While a diet that includes cruciferous vegetables is believed to promote health by activating cellular defense systems, their consumption following liver disease may be detrimental.

## Additional Information

**How to cite this article:** Yoda, E. *et al*. Isothiocyanates induce UGT1A1 in humanized *UGT1* mice in a CAR dependent fashion that is highly dependent upon oxidative stress. *Sci. Rep.* **7**, 46489; doi: 10.1038/srep46489 (2017).

**Publisher's note:** Springer Nature remains neutral with regard to jurisdictional claims in published maps and institutional affiliations.

## Supplementary Material

Supplementary Information

## Figures and Tables

**Table 1:** Primer sequences used for Reverse Transcription Quantitative-PCR (RT-qPCR).

Primer	Forward	Reverse
Human *UGT1A1*	5′-CCTTGCCTCATCAGAATTCCTTC-3′	5′-ATTGATCCCAAAGAGAAAACCAC3′-
Mouse *Cyp2b10*	5′-AAAGTCCCGTGGCAACTTCC-3′	5′-CATCCCAAAGTCTCTCATGG-3′
Mouse *Nqo1*	5′-TTTAGGGTCGTCTTGGCAAC-3′	5′-GTCTTCTCTGAATGGGCCAG-3′
Mouse *GstA1*	5′-AGCCCGTGCTTCACTACTTC-3′	5′-TCTTCAAACTCCACCCCTGC-3′
Mouse *GstA2*	5′-GAATCAGCAGCCTCCCCAAT-3′	5′-TCCATCAATGCAGCCACACT-3′
Mouse *cyclophiline*	5′-CAGACGCCACTGTCGCTT-3′	5′-TGTCTTTGGAACTTTGTCTGC-3′
Mouse *Akp3*	5′-CTGGAGCCCTACACCGACT-3′	5′-AGGCTTCTGGCGCTGTTAT-3′
Mouse *Sis*	5′-ACCCCTAGTCCTGGAAGGTG-3′	5′-CACATTTTGCCTTTGTTGGATGC-3′
Mouse *Krt20*	5′-CCTGCGAATTGACAATGCTA-3′	5′-CCTTGGAGATCAGCTTCCAC-3′
Mouse *Glb1*	5′-CACTGCTGCAACTGCTGG-3′	5′-ATGTATCGGAATGGCTGTCC-3′
Mouse *Lrp2*	5′-CCAGGATTCTGGTGATGAGG-3′	5′-CGGGAACTCCATCACAAACT-3′
Mouse *Nox4*	5′-TCTGGAAAACCTTCCTGCTG-3″	5′-CCGGCACATAGGTAAAAGGA-3′
