# Supplementary material for: Isothiocyanates induce UGT1A1 in humanized UGT1 mice in a CAR dependent fashion that is highly dependent upon oxidative stress
Source: Sci Rep. 2017 Apr 19;7:46489. doi: 10.1038/srep46489 (PMC5395973; doi:10.1038/srep46489)
Supplement: Supplementary Information [file srep46489-s1.pdf]

Supplemental Information

Title: **Isothiocyanates induce UGT1A1 in humanized *UGT1* mice in a CAR dependent fashion that is highly dependent upon oxidative stress**

Short title: **Cruciferous vegetables and activation of nuclear receptors**

Emiko Yoda [1,2], Miles Paszek[1], Camille Konopnicki[1], Ryoichi Fujiwara[3], Shujuan Chen[1] and Robert H. Tukey[1,*]

[1]Laboratory of Environmental Toxicology, Department of Pharmacology, University of California, San Diego, La Jolla, CA 92093.

[2]Division of Health Chemistry, Department of Healthcare and Regulatory Sciences, School of Pharmacy, Showa University, 1-5-8 Hatanodai, Shinagawa-ku, Tokyo 142-8555, Japan.

[3]Department of Pharmaceutics, School of Pharmacy, Kitasato University, 6-9-1 Shirokane, Minato-ku, Tokyo 108-8641, Japan.

Supplemental Figure 1

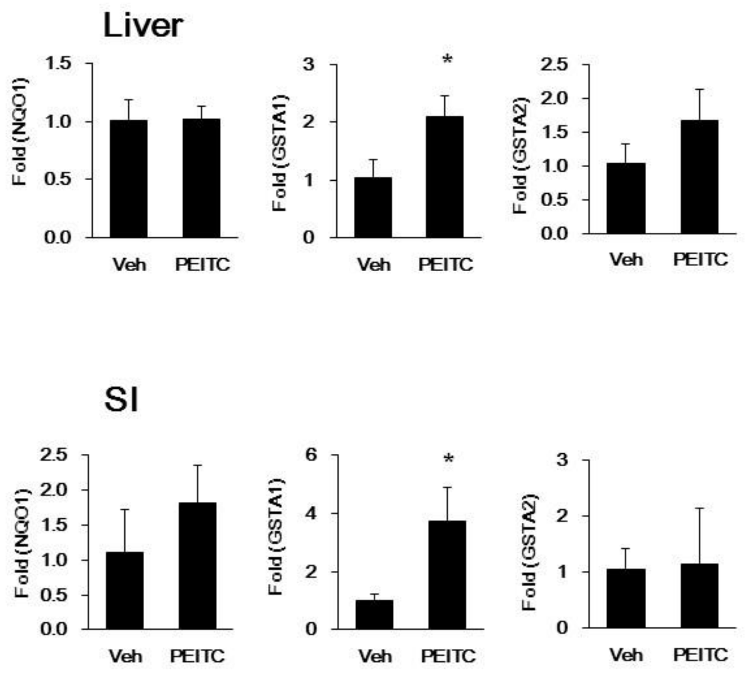

Supplemental Figure 1. *Analysis of Nrf2 target genes*. Using RNA isolated in Figure 1 (neonatal *hUGT1* mice), gene expression patterns for *Nqo1*, *Gsta1* and *Gsta2* were quantitated by RT-qPCR expressed as Fold induction. Values are the means of ± SD (n=3). Statistically significant differences between vehicle (Veh) and PEITC are indicated by asterisks (Student *t* test: *, *P*<0.05).

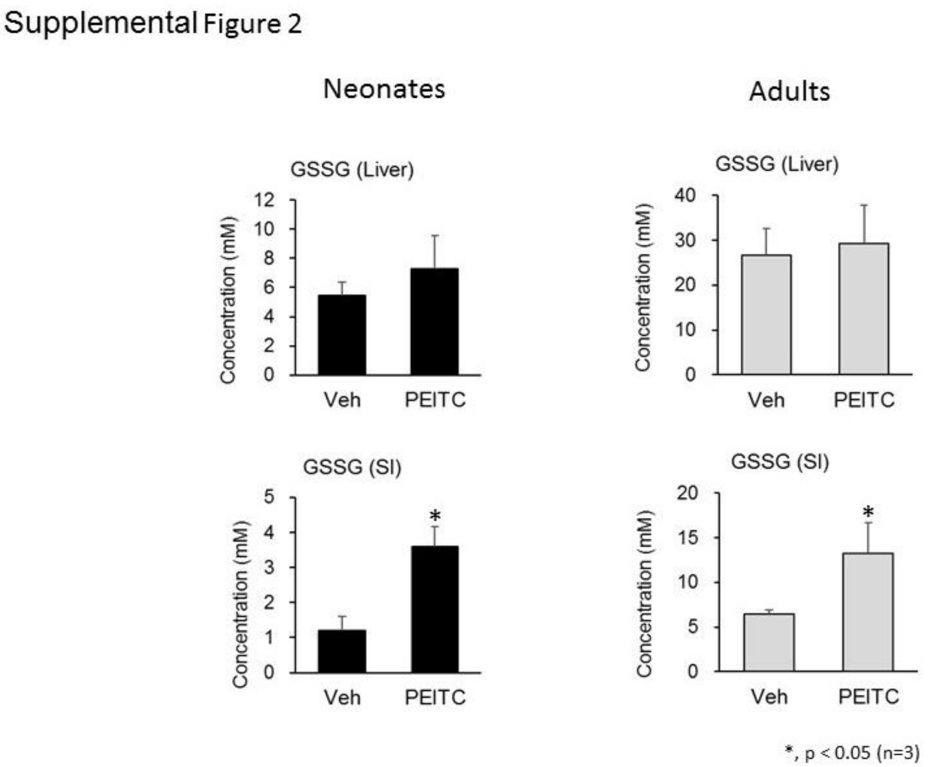

Supplemental Figure 2. *Oxidative stress following PEITC treatment*. **A**. Neonatal *hUGT1* mice were given an oral dose of PEITC (200 mg/kg) and GSSG levels measured in liver and small intestine (SI) after 24 hours. **B**. The same experiment was conducted on adult (6 week old) *hUGT1* mice. Values are the means of ± SD (n=3). Statistically significant differences between vehicle (Veh) and PEITC are indicated by asterisks (Student *t* test: *, P<0.05).

Supplemental Figure 3

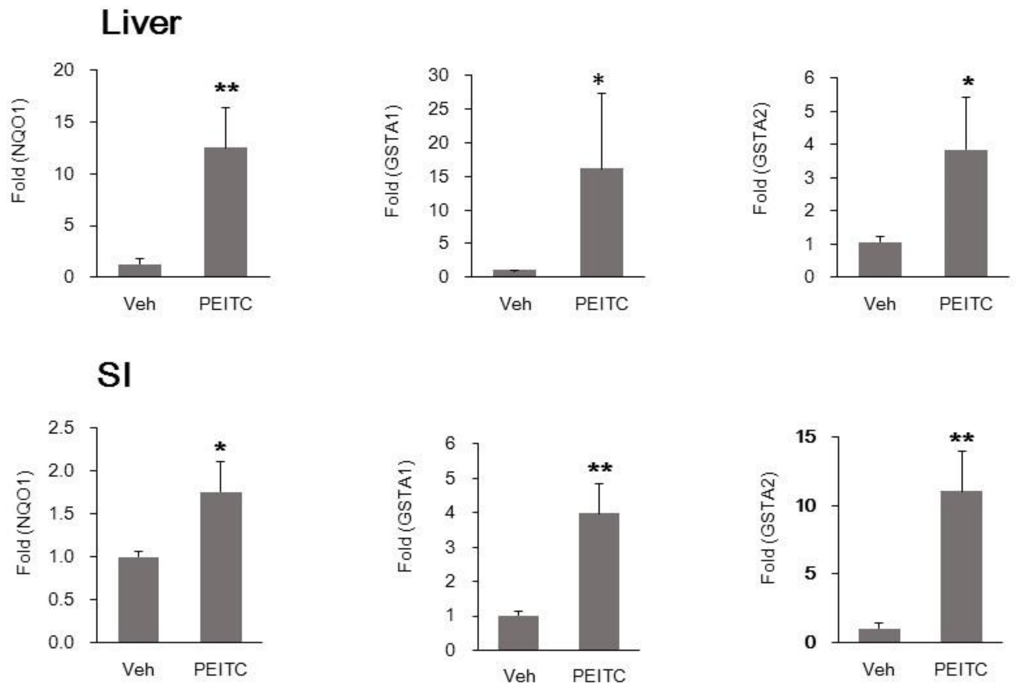

Supplemental Figure 3. *Impact of PEITC on Nrf2 target genes in adult mice*. RNA prepared from PEITC treated *hUGT1/Car*$^{+/-}$ and *hUGT1/Car*$^{-/-}$ mice from liver and small intestine (Figure 4) was used to quantitate *Nqo1*, *Gsta1* and *Gsta2* gene expression by real time PCR. Values are the means of ± SD (n>3). Statistically significant differences between vehicle (Veh) and PEITC are indicated by asterisks (Student *t* test: *, $P<0.05$; **, $P<0.01$).

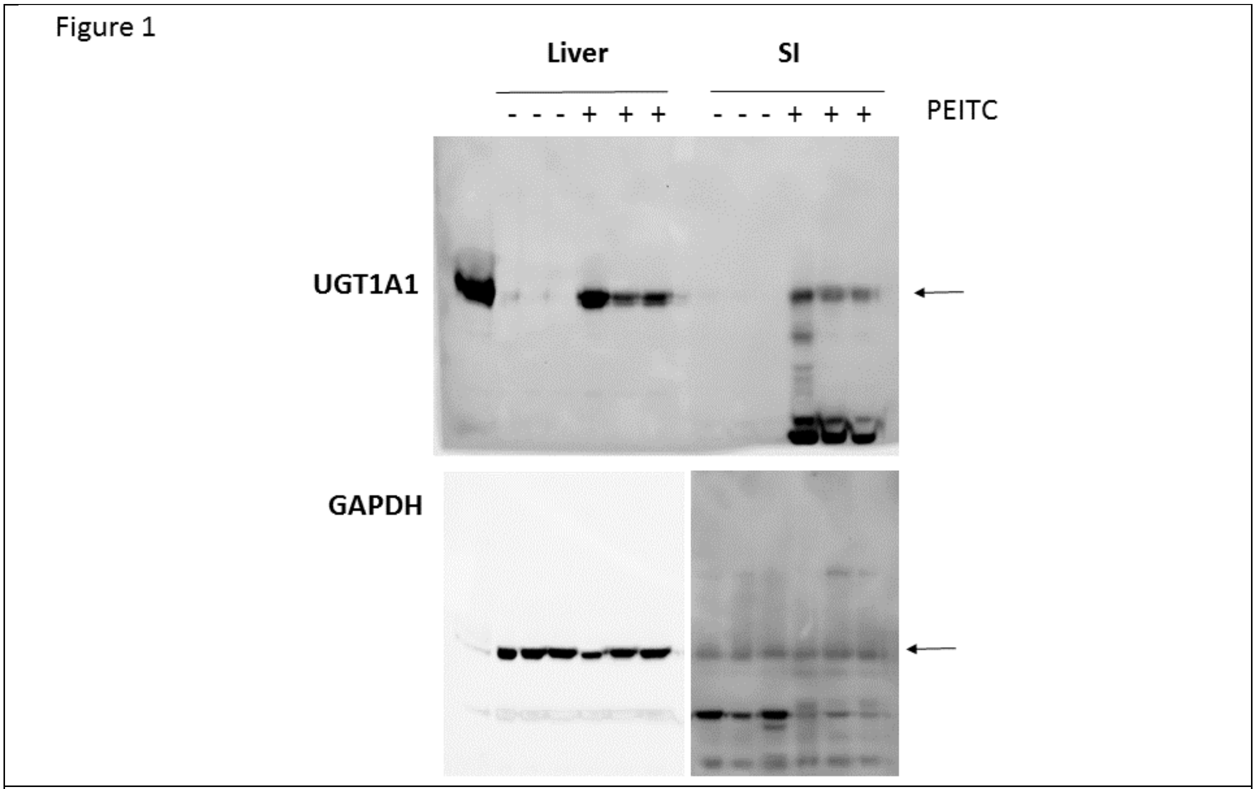

Supplemental Figure 4. Full length Western blot used for Figure 1.

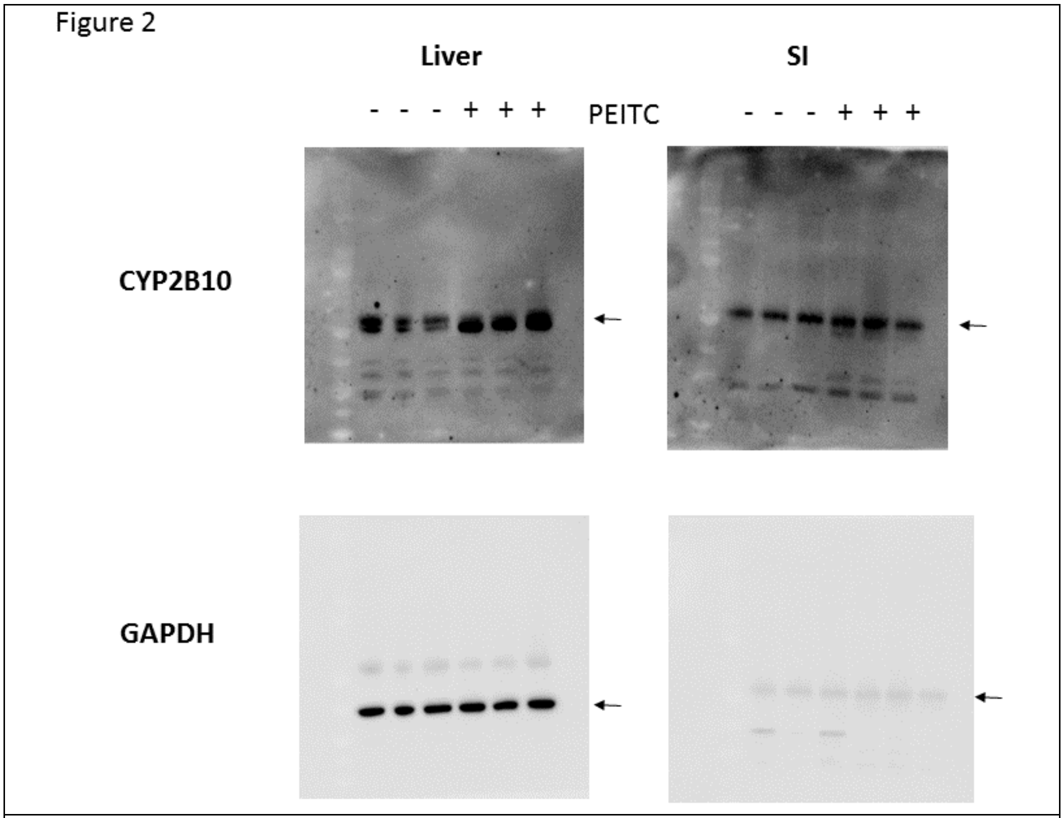

Supplemental Figure 5. Full length Western blots used for Figure 2.

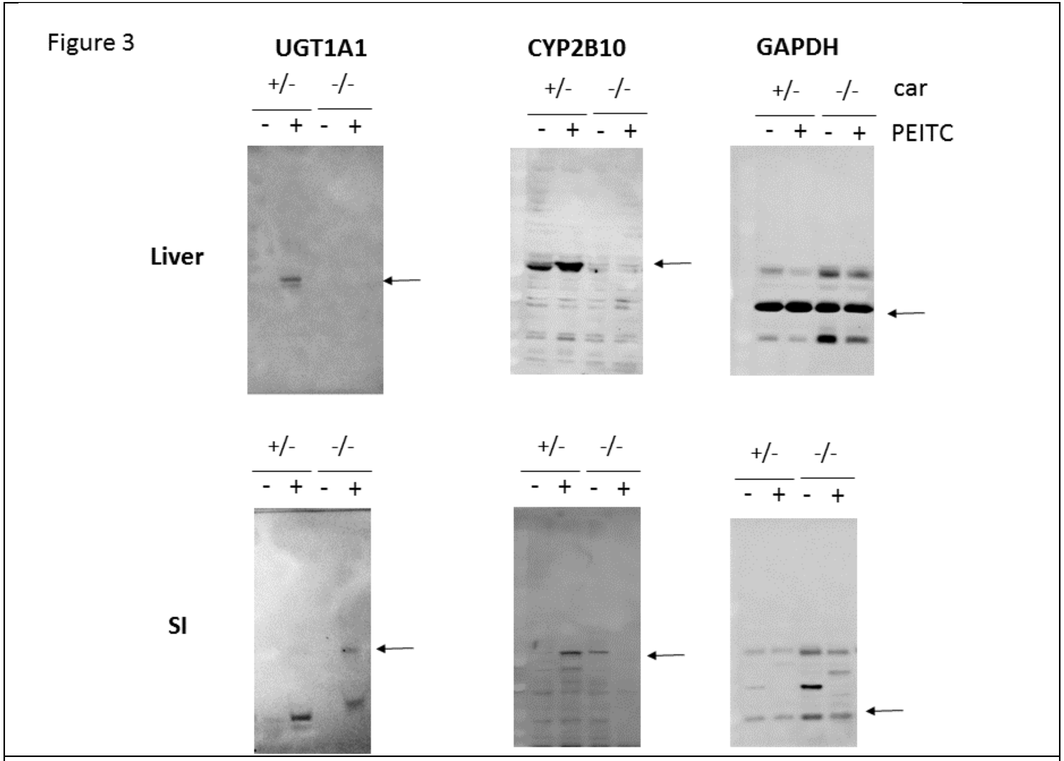

Supplemental Figure 6. Full length Western blots used for Figure 3.

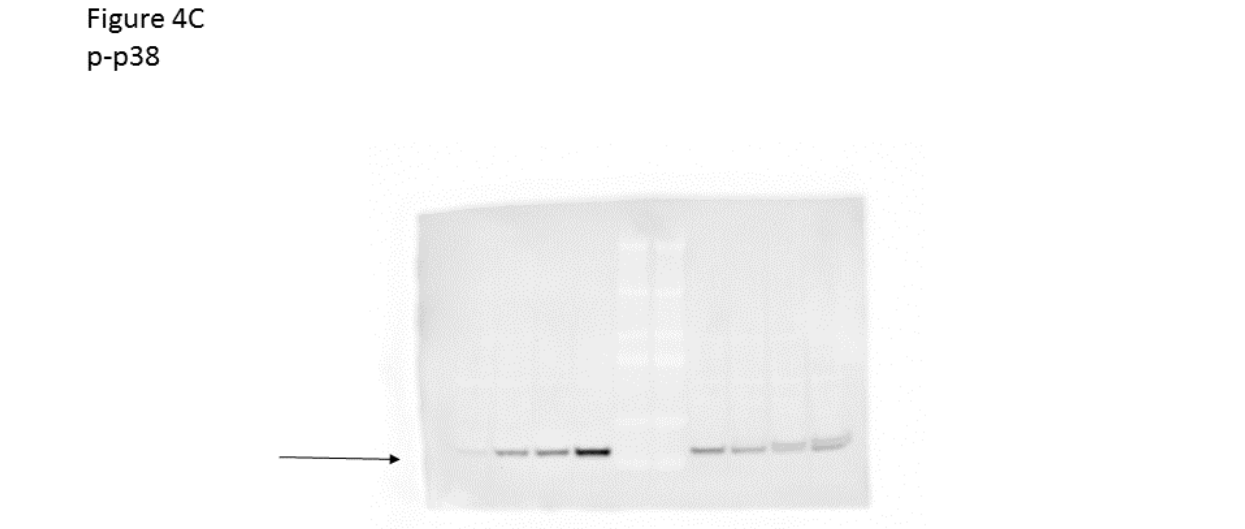

Supplemental Figure 7. Full length Western blot used in Figure 4

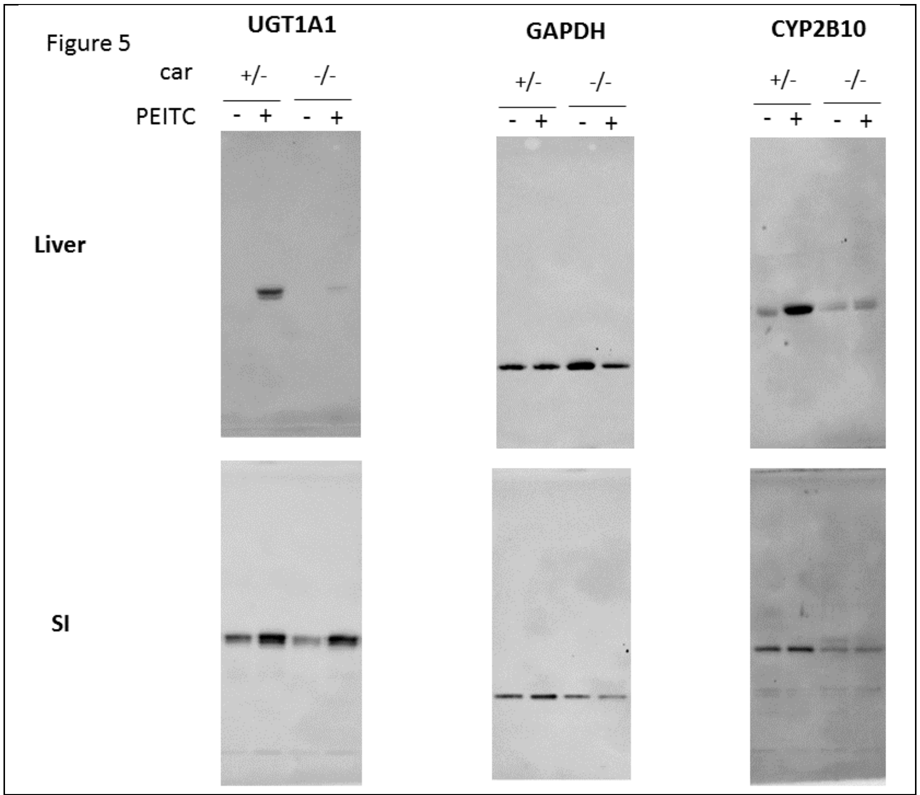

Supplemental Figure 7. Full length Western blots used for Figure 5.

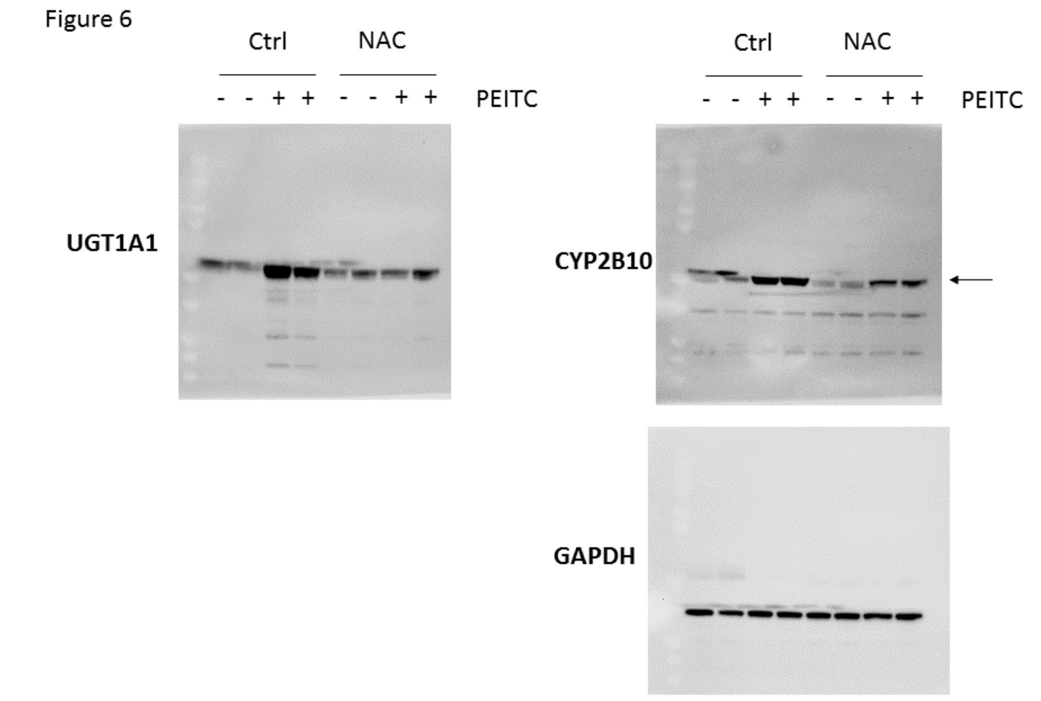

Supplemental Figure 8. Full length Western blots used for Figure 6.
